# Challenges during the second wave of COVID-19 in Brunei Darussalam: National Isolation Centre to National COVID-19 Hospital

**DOI:** 10.5365/wpsar.2022.13.3.913

**Published:** 2022-07-22

**Authors:** Noor Affizan Rahman, Muhammad Syafiq Abdullah, Rosmonaliza Asli, Pui Lin Chong, Babu Ivan Mani, Vui Heng Chong

**Affiliations:** a Department of Medicine, Pengiran Muda Mahkota Pengiran Muda Haji Al-Muhtadee Billah Hospital, Tutong, Brunei Darussalam.; b Department of Medicine, Raja Isteri Pengiran Anak Saleha Hospital, Bandar Seri Begawan, Brunei Darussalam.

## Abstract

**Problem:**

Soon after the start of the second wave of coronavirus disease 2019 (COVID-19) in Brunei Darussalam, which was confirmed to be due to the more infectious Delta strain of severe acute respiratory syndrome coronavirus 2 (SARS-CoV-2), it became apparent that the National Isolation Centre (NIC) was not coping.

**Context:**

The NIC was the only isolation and treatment centre for COVID-19 in Brunei Darussalam. During the first wave and the first 11 days of the second wave, all confirmed cases were admitted to the NIC for isolation and treatment in line with the management strategy to isolate all confirmed cases to control the outbreak.

**Action:**

The Ministry of Health opened five community isolation centres and two quarantine centres to divert asymptomatic and mild cases from the NIC. The community isolation centres also functioned as triage centres for the NIC, and the quarantine centres accommodated recovered patients who did not have their own quarantine facilities.

**Outcome:**

The community isolation and quarantine centres diverted cases from the NIC and enabled recovered cases to be transferred to these step-down facilities. This reduced the NIC’s occupancy to a safe level and enabled the reorganization of the NIC to function as a treatment centre and a national COVID-19 hospital.

**Discussion:**

During any disease outbreak, health facilities must be prepared to adapt to changing situations. Strong leadership, stakeholder commitments, teamwork and constant communication are important in this process.

## PROBLEM

As of 13 June 2022, the coronavirus disease 2019 (COVID-19) pandemic had amounted to more than 532 million cases globally and more than 6.3 million deaths. (*1*) Brunei Darussalam reported the first case of COVID-19 on 9 March 2020 and implemented measures that successfully contained the first wave, with the last case of community spread reported on 6 May 2020. (*2*) From then, Brunei Darussalam was at Level 2 out of the four levels of COVID-19 transmission classified by the World Health Organization, with no community spread and only sporadic imported cases. (*3*) While measures implemented during the first wave had remained in place to monitor the situation, it was not possible to accurately predict the impact of the second wave until it happened. Soon after the second wave started on 7 August 2021, owing to the more infectious Delta strain of the severe acute respiratory syndrome coronavirus 2 (SARS-CoV-2), it quickly became apparent that the existing quarantine and isolation measures would not be sufficient. A similar situation was faced by many countries where health-care systems were stretched or had collapsed due to factors such as overwhelming numbers of COVID-19 patients, burnout among health-care providers and the depletion of resources. (*4*-*6*)

With the expected increase in the number of COVID-19 cases, it was important to change the role of the National Isolation Centre (NIC) from an isolation centre to a COVID-19 hospital. This report describes the challenges faced by Brunei Darussalam during the second wave of COVID-19, the measures implemented to avoid overwhelming the NIC and its transition to a COVID-19 hospital.

## CONTEXT

### Setting

Brunei Darussalam, with an estimated population of 453 600 (2020), is divided into four districts, each served by a government hospital. The NIC is the designated national isolation facility for any infectious disease, located in the Tutong district, consisting of three wards: one intensive care unit (ICU) and two ICU-capable isolation wards with a total bed capacity of 27. Prior to COVID-19, this complex was used for isolation and treatment of pulmonary tuberculosis. It is located adjacent to the district government hospital, which has 135 beds in six wards. These two facilities have a combined bed capacity of 162.

When COVID-19 was discovered in Wuhan, the People’s Republic of China, in late December 2019, Brunei Darussalam began closely monitoring the situation, prepared thorough strengthening of surveillance and testing processes, and reviewed and updated infection control and outbreak management protocols. Prior to the first wave, several suspected cases, mainly travellers returning from affected areas, were isolated in the NIC, but all tested negative for SARS-CoV-2. When the first case of COVID-19 was detected, all non-essential services were closed at the NIC, and all inpatients were transferred to other hospitals in anticipation of more cases. The NIC and district hospital were converted into a COVID-19 isolation and treatment centre.

Due to the further increase in cases in the early phase, the Ministry of Health (MoH) decided to build the National Isolation Centre Extension (NICE) adjacent to the NIC. The NICE consisted of 20 bays with six to eight beds per bay, including an ICU-capable bay with six isolation rooms. However, soon after the NICE was completed, the first wave was brought under control. The district hospital eventually reopened for general services, and the original NIC cared for a small number of imported cases of COVID-19 until the second wave began. Altogether, the three complexes, henceforth all referred to as the NIC, increased bed capacity to between 260 (preferred) and 320 (maximum), taking into consideration the available personnel.

### Testing and triaging strategy

Over the course of the pandemic, the testing and management protocols used in the NIC were revised several times. (*7*) The current testing protocol requires testing by reverse transcription polymerase chain reaction (RT–PCR) on day 8 after the first positive RT–PCR (designated as day 0 of COVID-19 infection). If the test is negative, the patient is discharged to a designated quarantine centre. If the test is positive, testing is repeated every 48 hours until it is negative. The case must also be symptom-free or mildly symptomatic with no or resolving abnormalities in the investigations before discharge. Post-discharge cases are isolated for another 2 weeks, either at a designated isolation facility or at the case’s own accommodation, if suitable.

For risk stratification, cases were initially categorized by disease severity (mild, moderate, severe and critical) based on the clinical, laboratory and imaging parameters used in the first wave. (*8*) However, on  13 August 2021, during the second wave, an adjustment was introduced to the new categorization system, which was adapted from that used by the MoH Malaysia, (*9*) and based on clinical assessment supplemented by chest imaging. This system is more specific and facilitated the daily categorization of cases (**Table 1**).

**Table 1:** Clinical categories for adults with COVID-19, Brunei Darussalam

Staging	Description
**Category 1**	Asymptomatic
**Category 2**	Symptomatic without pneumonia^a^
**Category 3**	Symptomatic with pneumonia
**Category 4**	Symptomatic with pneumonia requiring supplemental oxygen
**Category 5**	Critically ill (respiratory failure requiring mechanical ventilation with or without other organ failure)
**Category 2** can be further divided into two subcategories: MILD (2A) and MODERATE (2B). This subcategorization is for clinical management team reference only to risk-stratify patients who can be transferred to the community isolation facility.	
**Category 2A (Mild)**	**Category 2B (Moderate)**
Sore throat or rhinorrhoea with no fever or dyspnoea	Persistent fever ((*3*)2 days) or new onset fever
Cough with no fever or dyspnoea	Exertional dyspnoea
Loss of taste but able to consume food orally	Chest pain
Loss of smell	Unable to consume food orally
Diarrhoea two times or less within 24 hours with normal urine output	Worsening lethargy, e.g. difficulty with usual activities or struggling to get out of bed
Nausea and vomiting with normal urine output	Unable to ambulate without assistance
Mild lethargy but still able to carry out daily activities	Worsening or persistent symptoms, e.g. cough, nausea, vomiting or diarrhoea
Myalgia but still able to carry out daily activities	Reduced consciousness
-	Reduced urine output in the last 24 hours

Source: adapted from Ministry of Health Malaysia guidelines. (*9*)

^a^ Determined by either clinical findings or chest imaging.

### Challenges

By day 7 of the second wave (13 August 2021), the preferred bed capacity of 260 was reached, and it was clear that the NIC would be quickly overwhelmed. The national number of new and cumulative confirmed cases recorded over the first 16 days reached the maximum occupancy threshold of 320 by day 9 (**Fig. 1a**). The number of new daily cases reached a peak of 314 on day 16 (22 August). Due to the cumulative increase, a backlog of patients waiting to be admitted for assessment was expected. Most of the cases admitted to the NIC were asymptomatic or mildly symptomatic (Categories 1 and 2) (**Fig. 1b**).

**Fig. 1a:**
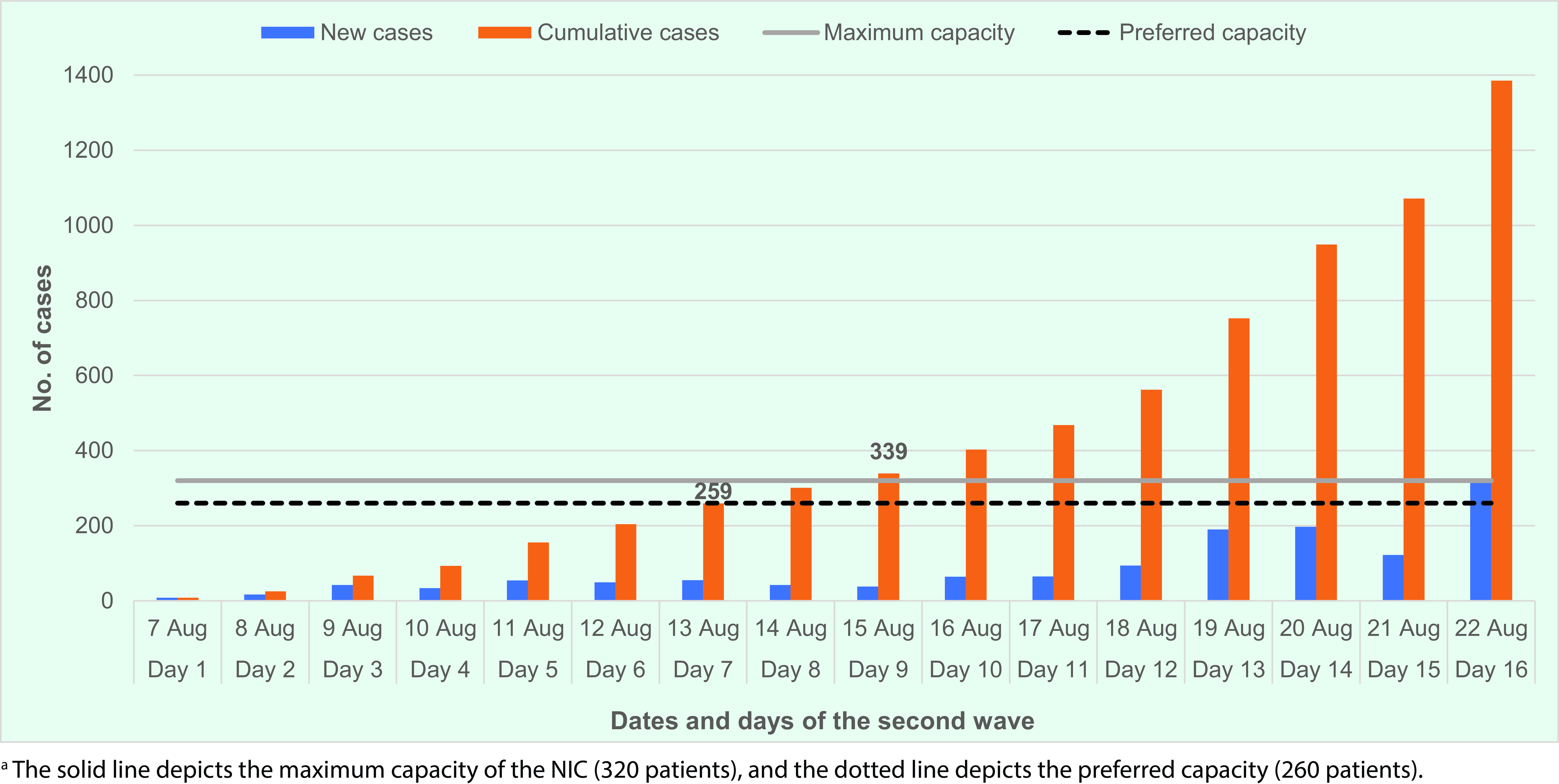
Recorded number of daily new and cumulative COVID-19 cases for the first 16 days of the second wave, Brunei Darussalam

[**insert** Figure 1a]

**Fig. 1b:**
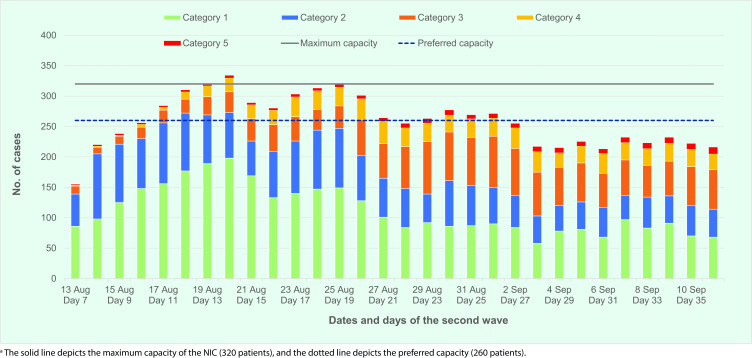
Recorded number of COVID-19 cases admitted to the NIC from 13 August (day 7 of second wave) to 11 September 2021 by clinical category, Brunei Darrusalam

[**insert** Figure 1b]

The situation was compounded by logistical, personnel and supply chain issues as a result of service expansion to cater to the increasing number of cases. Existing personnel had to be reallocated to areas of need, and additional personnel were sought from within the MoH, other ministries and volunteers to run the centre. Support services such as transportation, laundry, catering, safety and cleanliness had to be increased. Similarly, maintaining adequate stocks of consumables and medications and ensuring smooth supply chains were crucial.

It was also important to have sufficient numbers and the right mix of doctors and nurses to maintain services and manage cases with different medical conditions. Redeployment from other hospitals was required at short notice, further straining the personnel shortage in source hospitals. With rapidly increasing numbers of patients being admitted and spread out across the three buildings of the NIC, it was challenging to group cases with similar medical needs and levels of care together. In addition, the different social needs of patients needed to be taken into consideration.

## ACTION

### Opening of supporting community isolation and quarantine centres

An important step taken by the MoH was the identification and conversion of existing government facilities into isolation centres, which enabled the diversion of milder cases from the NIC. In total, there were seven centres, all of which were suitable government complexes for isolation, such as schools or training centres. Initially, there were two centres (Centres A and B) that received patients who remained positive for SARS-CoV-2 by RT–PCR (Centre A) on day 8 and patients who had recovered (RT–PCR negative on day 8 or 10) but did not have their own suitable accommodation for isolation (Centre B). Later, most of the centres also served as triage centres and admitted asymptomatic or mild cases for isolation (**Table 2**). The Emergency Medical Ambulance Service initially triaged patients and decided on their destination according to an admission criterion.

**Table 2:** Isolation and quarantine centres by role, category and occupancy as of 18 September 2021,  Brunei Darussalam

Centre	Facility	Role	Categories of cases admitted	Capacity (overall/ usable)	Current occupancy	Staffing personnel
National isolation centre	Isolation centres Hospital	Isolation and treatment centre	All categories	320/320	238	Existing and redeployed staff
Community isolation centre A	Youth national programme centre	Isolation centre	1, 2A	532/532	353	Onsite medical team Military security
Community isolation centre B	Secondary and boarding school	Isolation centre	1, 2A, 2B	789/511	618	Onsite medical team Military security
Community isolation centre C	Army battalion camp	Isolation centre	1	300/200	40	Remote medical team in Centre B Military security
Community isolation centre D	School	Isolation centre	1	320/320	79	Remote medical team based in district hospital Military security
Community isolation centre E	School	Isolation centre	1	222/222	10	Onsite military medical team Military security
Quarantine centre A	School	Post-discharge quarantine	Recovered	150/45	0	Administrative staff
Quarantine centre B	School	Post-discharge quarantine	Recovered	408/222	78	Administrative staff

### National Isolation Centre

The process of transforming the NIC from an isolation centre to a national COVID-19 hospital required several conditions to be met. The diversion of asymptomatic and mildly symptomatic cases to other isolation centres led to a reduction in total occupancy and allowed for personnel distribution to improve the nurse-to-patient and doctor-to-patient ratios. This also provided the opportunity to restructure and introduce other relevant specialty services to cater to the various medical needs of patients such as pregnant patients, patients with end-stage renal failure and patients needing intensive care and monitoring.

### Home isolation

Due to the increase in the number of patients waiting to be admitted to isolation centres, home quarantine/isolation was attempted. This had to be discontinued due to patients breaking quarantine orders and difficulties with monitoring adherence. Since then, all cases are required to stay in designated isolation centres.

## OUTCOMES

With the opening of the community isolation and quarantine centres, the number of cases admitted to the NIC declined with the opening of Centre A (which started accepting patients on 18 August 2021) and declined further from day 20 with the opening of Centre B, evident from day 15 (21 August 2021) (**Fig. 1b**). This also coincided with the increase in Category 3 and 4 patients admitted to the NIC, highlighting the importance of the new centres. With the reduction in occupancy at the NIC, it was possible to carry out the restructuring and adjustment of processes to function as a COVID-19 hospital.

Restructuring of the isolation processes allowed for increasing the capacity of high-dependency units and ICUs, converting existing wards into a dedicated obstetrics and gynaecology (OB/GYN) ward with en-suite labour room, paediatric ICU and neonatal ICU, expanding physiotherapy services particularly chest physiotherapies, increasing dialysis points, and establishing a remote on-call surgical team based in the main tertiary hospital located in the capital that was ready for acute surgical emergencies (**Table 3**). These changes also allowed for grouping of patients with similar medical needs (i.e. obstetric, renal dialysis and paediatric patients), effective allocation and distribution of nurses and doctors according to areas of expertise and improvements in patient care.

**Table 3:** List of medical services available before and during the COVID-19 outbreak

Specialties	Before COVID-19	Changes during COVID-19	Description
Intensive care/ High-dependency setting	Services not available 9 capable rooms 18 capable rooms	Increased capacity of ICU and high-dependency bay	27 ICU-capable rooms, 24 high-dependency beds
Nephrology	Services not available 2 capable rooms (2 dialysis points)	Increased dialysis capability	Increased to 16 dialysis points
OB/GYN	Outpatient clinics only Unused OB/GYN ward/labour room	Reopening of the ward with labour room	15 beds
OT	2 ready for use for minor cases 1 unused and ready for use	To operationalize OTs 1: OB/GYN ward 2: For other cases	Operationalization of all OTs
PICU and NICU	Services not available	Initially used NICE isolation rooms as designated PICU and NICU isolation rooms due to their high-dependency readiness Later relocated to be near the OB/GYN ward	Conversion of a ward near the OB/GYN ward
Surgery	Outpatient clinics Day-case surgery	Remote consultant on-call Team ready for acute surgical emergencies Available junior surgeons	Team on-call from another hospital
Physiotherapy service	Visiting services that were stopped during COVID-19	Re-introduction of regular physiotherapy, especially chest physiotherapy	Team of physiotherapists 5 days a week

NICU: neonatal intensive care unit; OB/GYN: obstetrics and gynaecology; OT: operating theatre; PICU: paediatric intensive care unit.

## Discussion

In any disease outbreak, it is important that the health-care system is prepared for the unexpected. A key lesson learned from the ongoing COVID-19 pandemic is that preparedness is very important. Even then, it is not always possible to predict whether preparations will be adequate to cope with demand. Our experience highlighted this when the NIC was almost overwhelmed, but this was averted by the opening of community isolation and quarantine centres so that asymptomatic or mild cases could be diverted away from the NIC. This also enabled the NIC to restructure and transition from an isolation centre to a COVID-19 hospital to deal with the increasing numbers of more severe cases.

Our success was dependent on the system’s ability to rapidly adapt to changing situations. It also required strong leadership, stakeholder commitment, teamwork, and especially constant and open communication between all stakeholders. Since the start of the second wave, daily online conferences were scheduled between the different centres and the MoH. These conferences were headed by the Minister of Health or executive-level officials, allowing for rapid decision-making. Involvement of the other ministries made it possible for the use and conversion of other facilities into isolation centres, and enabled provision of security by the armed forces. Centres where medical teams monitored patients remotely were under the control of the armed forces. Therefore, good teamwork within and between the MoH and other agencies was essential. Similar to during the first wave, there was considerable goodwill from the public and organizations, with donations in the form of food and daily-use items for the staff and patients.

Our experience highlighted that even during ongoing major disease outbreaks, it is possible to restructure services to cater to the changing situations and needs of cases. The transition of the NIC from an isolation and treatment centre to a COVID-19 hospital was made possible through strong leadership and commitment of relevant stakeholders.
